# Impact of Some Ecological Factors on Fecal Contamination of Drinking Water by Diarrheagenic Antibiotic-Resistant* Escherichia coli* in Zagazig City, Egypt

**DOI:** 10.1155/2016/6240703

**Published:** 2016-09-20

**Authors:** Ahmed Elsadek Fakhr, Maha Kamal Gohar, Amal Hassan Atta

**Affiliations:** Microbiology and Immunology Department, Faculty of Medicine, Zagazig University, Zagazig, Egypt

## Abstract

Fecal contamination of drinking water is a major health problem which accounts for many cases of diarrhea mainly in infants and foreigners. This contamination is a complex interaction of many parameters. Antibiotic resistance among bacterial isolates complicates the problem. The study was done to identify fecal contamination of drinking water by Diarrheagenic Antibiotic-Resistant* Escherichia coli* in Zagazig city and to trace reasons for such contamination, three hundred potable water samples were investigated for* E. coli* existence. Locations of* E. coli* positive samples were investigated in relation to population density, water source, and type of water pipe. Sixteen* E. coli* strains were isolated. Antibiotic sensitivity was done and enterotoxigenic, enteropathogenic, and enterohaemorrhagic virulence genes were investigated by PCR. Probability of fecal contamination correlated with higher population density, with increased distance from Zagazig water plant, and with asbestos cement water pipes. Resistance to at least one antimicrobial drug was found in all isolates. Virulence genes were detected in a rate of 26.27%, 13.13%, 20%, 6.67%, and 33.33% for LT, ST, stx1, stx2, and eae genes, respectively. This relatively high frequency of fecal contamination points towards the high risk of developing diarrhea by antibiotic resistant DEC in low socioeconomic communities particularly with old fashion distribution systems.

## 1. Introduction

Waterborne diseases are a significant public health issue, and many originate from contact with water contaminated with human fecal material [1]. Each year, more than 800,000 children younger than 5 years of age die from diarrhea, mostly in developing countries, which accounts for 11% of children deaths under the age of five years [2].

There are over 100 different types of bacteria, protozoa, and viruses that can be transmitted through contaminated water with numerous outbreaks which have been reported. Diarrheagenic* E*.* coli* (DEC) are among the most common etiologic agents of diarrhea from water origin. They are classified according to their virulence factors and other phenotypic traits into enterotoxigenic* E*.* coli* (ETEC), enteropathogenic* E*.* coli* (EPEC), Vero toxin/Shiga toxin-producing* E*.* coli* (VTEC/STEC) or enterohaemorrhagic* E*.* coli* (EHEC), enteroinvasive* E*.* coli* (EIEC), diffusely adherent* E*.* coli* (DAEC), and enteroaggregative* E*.* coli* (EAEC). All are known to be endemic in essentially all developing countries [3].

Similar to many other diarrheal diseases, ingestion of contaminated food and water can result in diarrhea due to DEC [4, 5]. Whenever inadequate drinking water and poor sanitation are found, DEC represent a major cause of diarrheal illness. Different sources of waters including drinking water, rivers, swimming pools, surface waters, and lakes have been found to harbor these organisms [6] and transmission can occur while drinking, bathing, and/or using water for food preparation [7]. Among the six known Diarrheagenic* Escherichia coli* ETEC, EHEC and EPEC are the most significant, with higher impact on countries of the developing world [8].

ETEC is the one which can produce heat labile and/or heat stable enterotoxins in addition to colonization factors which enable the organisms to colonize the small intestine and make diarrhea. Both enterotoxins and colonization factors differentiate ETEC from other categories of Diarrheagenic* E*.* coli* [7]. EPEC is known as a main cause of diarrhea among children less than 2 years of age particularly in the developing world. It can be defined as intimin (encoded by* eae* gene) containing* E*.* coli* bacteria that have the ability to form the distinctive histopathology attachment and effacing (A/E) lesions on intestinal cells but do not own the stx gene.

Shiga toxin-producing/enterohaemorrhagic* E*.* coli* (STEC/EHEC) or Vero toxin-producing* E*.* coli* (VTEC) was recognized in 1983 as a cause of hemorrhagic colitis (HC) with a recent large outbreak identified in Germany [9, 10].

According to the water-quality standards provided by WHO, the number of* E*.* coli* or thermotolerant bacteria should be nil in a 100 mL drinking water sample [11]. However, some standards allow the existence of ten coliforms/100 mL potable water [12]. Existence of coliform bacteria within a distribution system is a multifaceted interaction of physical, chemical, processing, and manufacturing aspects [13]. Distance from treatment place [14, 15], type, leaks, and breaks in the water pipe systems [16, 17], seasonal variation [18], and other environmental factors [19] were investigated and many were proved to have an influence on drinking water fecal contamination.

To monitor procedure of water treatment and disinfection, the total bacterial or heterotrophic plate counts can be useful. In addition, it can be used to verify the integrity of distribution systems, cleanliness, and existence of biofilms [18].

**Figure 1:**
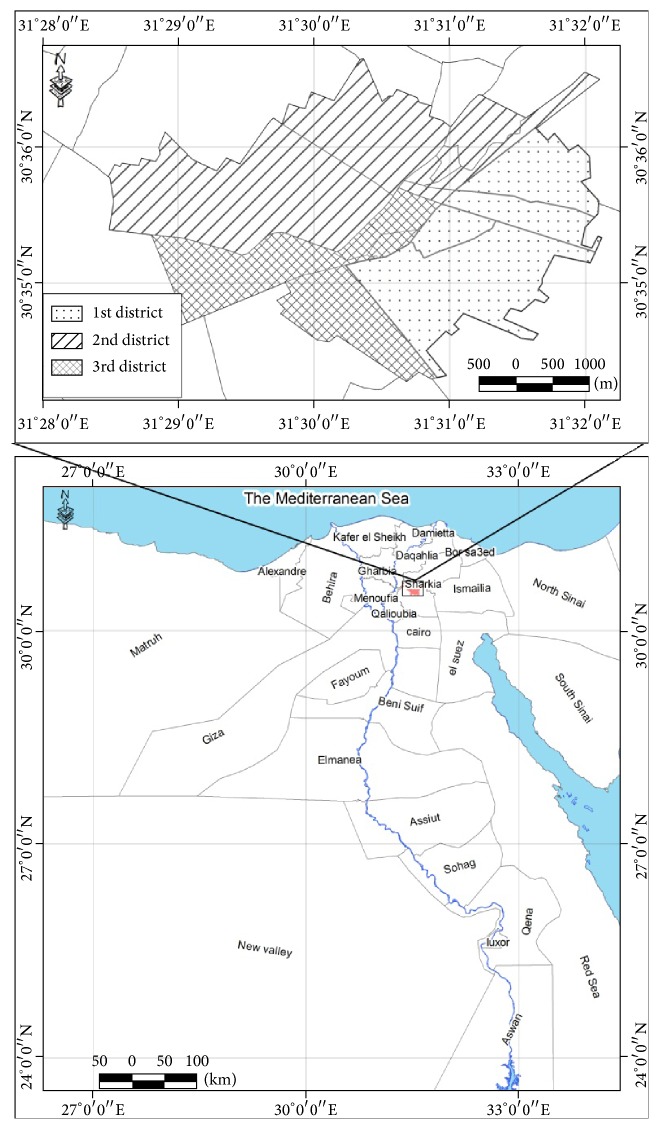
Location of Zagazig city in Arab Republic of Egypt (ARE) and its 3 districts.

Zagazig city (Figure 1) is the capital of Sharkia Governorate, located 100 km north east to Cairo and one of the biggest cities in Egypt in terms of population. The city is supplied by drinking water from different sources including surface and ground water. The old water distribution and sewage disposal systems in addition to poor sanitation facilities are frequent in communities like Zagazig. This may predispose to spread of fecal bacteria to surface and drinking water and hence lead to outbreaks of diarrhea in such communities.

In addition, drinking water pollution by fecal coliform bacteria harboring resistance genes is a water-quality issue of national scope and importance.* E*.* coli* can be considered as an important vehicle for the spread of resistance genes due to its plenty in such environments with a high risk of transfer from environment to human pathogens [20].

The objectives of this work were to determine the prevalence and reasons of fecal contamination of drinking water in Zagazig city, to determine the antibiotic resistance profile of the isolated* E*.* coli* and to recognize the presence of some Diarrheagenic* E*.* coli* (DEC) virulence genes among these isolates.

## 2. Material and Methods

**Figure 2:**
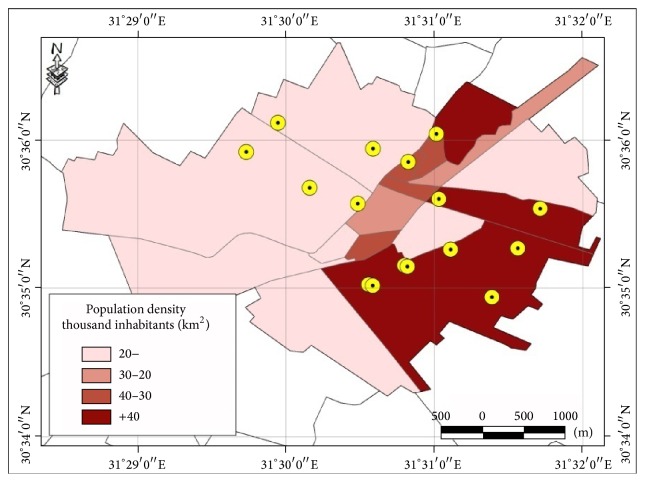
The positive* E*.* coli* isolates in relation to population density of different regions of Zagazig city.

Zagazig city is divided into 3 governmental districts (Figure 2). A total of 300 potable water samples were collected over the year 2011 from different sites chosen randomly among the mentioned previous districts, with respect to the population of each district. In addition, water samples from the main water plant and underground wells in the city were collected before entering the distribution system.

Maps for districts, water sources, population density, and water distribution system network of different regions in city were made according to data provided by Geographical Information System of Potable water and Sanitation Company of Zagazig city.

Tap water samples were aseptically collected and checked for fecal contamination by* E*.* coli* as described by Ram et al. [21]. Membrane filtration technique was used for one hundred (mL) volume from each site. Each membrane filter was aseptically cut into four pieces, transferred into a 25 mL MacConkey broth, and incubated for 24 hr. Subculture was done on eosin methylene blue (EMB) agar. Presumptive* E*.* coli* colonies with blue black metallic sheen on EMB were selected and confirmed as* E*.* coli* using routine biochemical assays.

### 2.1. Antimicrobial Susceptibility Determination

Isolated* E*.* coli* were tested against 8 antibiotics from six classes: *β*-lactams (ampicillin, 10 *μ*g/disc); aminoglycosides (amikacin, 10 *μ*g/disc; gentamicin, 10 *μ*g/disc); cephalosporins (cefotaxime, 30 *μ*g/disc); fluoroquinolones (ciprofloxacin, 5 *μ*g/disc; norfloxacin, 10 *μ*g/disc); phenicols (chloramphenicol, 30 *μ*g/disc); and tetracyclines (tetracycline, 30 *μ*g/disc). These tests were performed using an agar-diffusion method following Clinical and Laboratory Standards Institute breakpoints [22].

### 2.2. Virulence Genes Detection

For the determination of virulence genes, genomic DNA from* E*.* coli* isolates was extracted using G-spin Genomic DNA Extraction Kit (Bacteria) (iNtRON Biotechnology) following manufacturer's protocol. Bacterial total DNA from isolates was prepared for PCR by boiling technique as previously described by Zhao et al. [23]. Virulence genes of ETEC, EPEC, and EHEC were screened using the primers described by Ram et al. [24]. PCR primer sequences, their related gene targets, and expected amplification products size are shown in Table 1.

For amplification Maxime PCR PreMix kit (i-Taq) (iNtRON Biotechnology) was used. A typical 20 *μ*L PCR reaction assay mixture was accomplished by addition of 0.4 *μ*M from each primer, 4 *μ*L template DNA to the premix tubes containing 2.5 units of Taq DNA polymerase, 1x reaction buffer 2.5 mM of each dNTP, and 1x gel loading dye. Distilled water was added to bring the final volume to 20 *μ*L.

PCR amplification was performed in Biometra thermal cycler T3000 (Biometra, Westburg BV, Netherlands). Using specific primers, genomic DNA was used for PCR amplification of stx1, stx2, and eae A genes where total DNA was used for LT1 and ST1 genes with the following cyclic conditions (stx1: 94°C min, 48°C 1 min, and 72°C 1 min; stx2: 94°C 1 min, 45°C 1 min, and 72°C 1 min; eae A: 94°C 1 min, 44°C 1 min, and 72°C 1 min; LT1: 94°C 1 min, 42°C 1 min, and 72°C 1 min; and ST1: 94°C 1 min, 49°C 1 min, and 72°C 1 min). PCR products were visualized and analyzed using agarose gel electrophoresis under UV illumination.

Statistical analysis was done using SPSS (Version 20.0). Intergroup comparison was performed using Chi-square test. *P* value < 0.05 was considered statistically significant.

## 3. Results

In present study, a total of 90 public water supply samples from the 1st district of Zagazig, 120 from the 2nd district, and 90 from the 3rd district, in addition to water samples before entering the distribution system were taken. All water samples were analyzed by MFT and subcultured on MacConkey broth and EMB for isolation of coliform and* E*.* coli*.

Total coliform count was nil in all water samples collected before joining the distribution system from all sources.

In the 1st district, 20 (22.2%) water samples were found to be contaminated by coliform bacilli. Out of these 9 (10.0%) were found to harbor* E*.* coli*. In the 2nd district, 16 (13.3%) water samples were found to be contaminated by coliform. Out of these 7 (5.83%) were found to harbor* E*.* coli*. In the 3rd district, no coliforms were obtained.

The percentage of noncoliform isolates ranged from 6.66% in the third district to 18.3% in the second district (Table 2).

There was an increase in incidences of total coliform (*P* = 0.05) and* E*.* coli* (*P* = 0.03) isolates in spring months with peak in March (Table 3).

By studying the maps for population density of the city, it was found that 11 (68.75%)* E*.* coli* were isolated from areas with highest population density. Only 3 samples (18.75%) were isolated from areas of intermediate density and 2 samples (12.5%) from lower population density (Figure 2).

**Figure 3:**
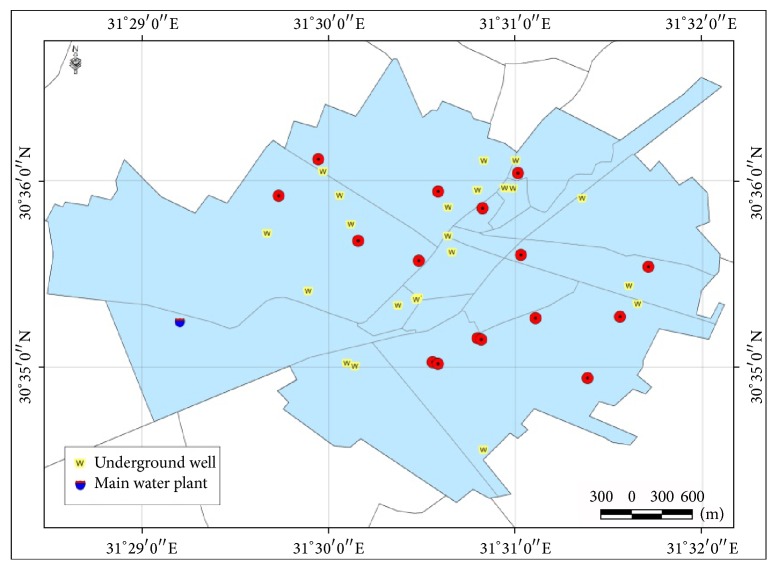
The positive* E*.* coli* isolates in relation to water sources in different regions of Zagazig.

It was observed that all isolates were away from the main water plant in the western area of Zagazig (Figure 3).

**Figure 4:**
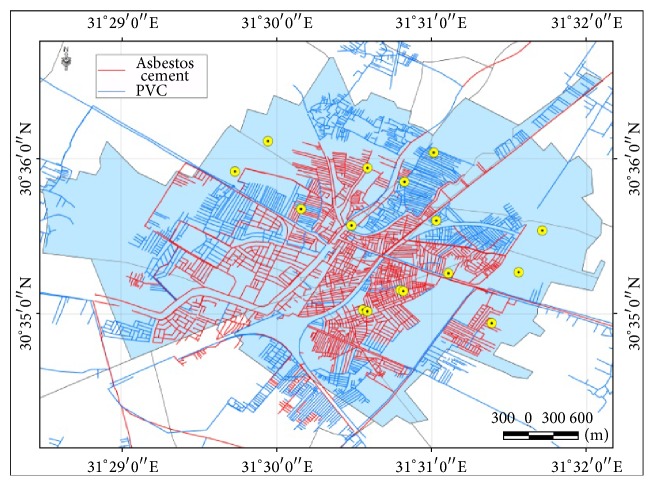
The positive* E*.* coli* isolates in relation to Zagazig water network with its different materials of construction.

The main types of water distribution peripheral system in Zagazig are the polyvinyl-chloride plastic type (PVC) and the asbestos cement types. It can be noticed through this map that 10 positive samples for* E*.* coli* fall in the area of the asbestos cement type. Two samples fall in the area of PVC pipes. Four samples fall in areas with nonclarified type of pipes (Figure 4).

In this study 10/16 (62.5%) of* E*.* coli* exhibited resistance to three or more antimicrobial agents.

*E*.* coli* isolates showed high resistance to cefotaxime and tetracycline (62.5%) followed by ampicillin (50%). Reduced susceptibility was observed with chloramphenicol (37.5%), followed by gentamycin (25%), norfloxacin (12.5%), and cefotaxime (12.5%). Highest sensitivity was found with amikacin (75%), gentamycin (75%), and norfloxacin (75%) followed by ciprofloxacin (62.5%).

As regards virulence genes detection by PCR, eight isolates (50.0%) were found to harbor at least one virulence gene. Four isolates had (LT) gene; 2 of them carry (ST) gene. Three* E*.* coli* isolates were found to have (stx1); one of them carries (stx2) gene as well. Five isolates carried eae A gene (Table 4).

## 4. Discussion

Globally, near 800 million people have no access to improved water sources and about 2.5 billion people do not have access to satisfactory sanitation [25]. Outbreaks of waterborne diarrheal diseases still have a serious health threat worldwide.

A better knowledge of the prevalence, ecology and distribution of fecal contamination in drinking water sources could be an important start for development of strategies to reduce the associated public health risk.

Zagazig city is the capital of Sharkia Governorate and one of the biggest cities in Egypt in terms of population which is near 500,000 with a population density over 60,000 inh./km^2^ in many of its regions.

The city has two sources of drinking water, surface and ground water. The chief supply of drinking water is Zagazig water plant located in the western area (3rd district). However, the increasing population of the city exceeds the capacity of the station and necessitates the assistance by a number of ground water wells. About 22 ground water wells are working now in the city and responsible for about 50% of the city water needs (Figure 3).

As the networks of water pipes in Zagazig are continuous, it was difficult to determine which regions are supplied by each well or which is supplied solely by the main station.

Total coliform prevalence among the 300 tap water samples taken from the city was 12%. Out of them 5.33% were* E*.* coli*. This was less than the prevalence detected by Ozgumus et al. [20] who found that 30% of their tap water samples isolated from coastal region of Northern Turkey are contaminated by coliform bacteria. This may be attributed to the larger study area which included rural and agricultural regions unlike our study which was restricted to the city.

In another study in India, Patel et al. [26] found that all the potable water samples exceeded the standard permissible limits. They explained this high prevalence by rainy environment in the area of study which saves warm humid conditions supporting microbial growth.

The interesting point is that a highly significant difference in the prevalence of total bacterial isolates, total coliform, and* E*.* coli* between the three districts of Zagazig was found (*P* value = 0.001, 0.004, and 0.06, resp.) with the least prevalence recorded in the 3rd district. Reasons for such finding were traced and many explanations were suggested.

First, the 3rd district is the one in which the main water plant is found, which makes most areas in this district supplied mainly by treated water from the plant with less chance to be contaminated at its way. Also in the areas near the main water plant the concentration of terminal chlorine is the highest; therefore* E*.* coli* which could contaminate this water are nonviable or sublethally injured and may not be detected by culture on selective media; however they are metabolically active [27].

Second, the 1st district which had the highest prevalence of pollution is the most far from the main plant water station as shown in (Figure 3). Moreover, this district is the area of highest population density that makes the individual share of services including safe water supply and sewage disposal is less and hence decreases the quality of these services (Figure 2).

A similar finding was noticed in an Egyptian study by Abu-Elyazeed and his colleagues, where the incidence of* E*.* coli* diarrhea increased in household crowding and decreased with the presence of sanitary latrine [28].

Also, Cheesbrough documented that the larger the population served, the longer the distribution system and therefore the greater the risk of contamination [29].

Third, the city has 2 main types of water pipes (Figure 4): the asbestos cement pipes and the polyvinyl-chloride plastic (PVC) type. The asbestos cement type is the one which was used for more than 30 years but now it is replaced with PVC for its known health risks. It was noticed that most of* E*.* coli* isolates were detected from sites where the asbestos cement type is the most prevalent. This could be explained by a 2012 study that illustrated that PVC has a lower water main break rate than cast iron, ductile iron, steel, asbestos cement, and concrete [16]. Also, the life span of asbestos cement type was exceeded or about to be exceeded in many of the city regions what makes it more susceptible for breakage and leaks. The surrounding environment could also age the asbestos cement pipes prematurely and increased the rate of leaks and bursts [17].

The total bacterial isolates were 30 out of 90, 38 out of 120, and 6 out of 90 in the 1st, 2nd, and 3rd districts, respectively. This may indicate that water from 3rd district has better treatment, disinfection, and integrity of distribution systems than the 1st and 2nd.

Although 3 samples were taken from each site at different times among the year,* E*.* coli* was isolated twice from only two of these sites. This means that contamination was an accidental event that is affected by different factors [13]. Absence of* E*.* coli* growth on repeating of sample from the same site can be attributed to either interference by the presence of high numbers of background heterotrophic bacteria or the inability of membrane filtration technique (MFT) to recover stressed or injured coliforms [30].

With regard to seasonality, the 300 water samples were taken all over the year but it was noticed that there is significant increase in the incidence of positive coliforms and* E*.* coli* samples in the spring with peak in March (Table 3). This is also matching with findings reported that DEC diarrhea and asymptomatic colonization are most common during warm periods of the year [28, 31, 32].

It was also reported that DEC contamination remains endemic all year with two peaks, one at the spring and the other in the autumn months [33, 34]. As the temperature rises when spring begins after winter months, bacterial growth increases and this continues in the summer months [35]. Such seasonality could be initiated by atmospheric temperature and extended by other ecological factors.

However, in this study the continuity of this high incidence in summer months was not observed. This may be attributed to the relatively dry nonrainy nature of summer climate in the location of study unlike that found in areas of other studies which have humid rainy climate in this season.

Increased utilization of antimicrobial agents in farming and animal husbandry, beside its use in medical therapy, has resulted in selective stress on environmental bacteria [36]. Therefore antibiotic resistance grows to be a notable problem in bacteria from aquatic environments including drinking water [37–40].

In this study, all of the isolated* E*.* coli* (*n* = 16) were resistant to at least one antimicrobial agent and 10 out of the 16 samples (62.5%) displayed resistance to three or more antimicrobial agents. This incidence of multidrug-resistant strains is near to that detected by Ram et al. [24] who found that 57.8% of* E*.* coli* strains isolated from surface water samples were multidrug-resistant. However, a relatively lower incidence was reported by Toroglu et al. [41], who found that 40% of the coliforms isolated from water samples in Turkey were multidrug-resistant.

Almost similar resistance pattern was found by Abdallah [42] in Egypt. The high levels of resistance to ampicillin were also in general agreement with many other studies [20, 43, 44].

However, unlike our results, Ozgumus and his colleagues [20] found that resistance to chloramphenicol was detected at low level (3%), probably resulting from limited use of this drug in veterinary or human medicine there.

The situation was different in a study done by Sayah et al. [45] in Red Cedar watershed in Michigan, USA. Among 26 strains of* E*.* coli* isolated from surface water, all of them were sensitive to all tried antibiotics except for one of the cephalosporin to which 80.6% of isolates were resistant.

This variation in resistance pattern between different countries reflects human influence on the environment as it depends on the extent of consumption of a certain antibiotic in a community either in humans, in animals, or in agriculture. This also should pay our attention to the hazard of nonrationalized use of antibiotics which threatens us with an era of noneffective therapy against bacteria.

The relative extent of* E*.* coli* virulence genes appears to vary from one geographic area to another in environmental samples, asymptomatic carriers, or from diarrheal patients. Virulence genes are seen as perfect targets to distinguish the pathogenic potential of* E. coli* isolate [46]. However, the ability of these isolates to cause human diarrheal illness could not be established as this requires proper combination of virulence genes to cause disease [47].

In this study LT, ST, stx1, stx2, and eae genes were investigated among the isolated* E*.* coli* strains. eae gene was the one most frequently found (33.33%) followed by 26.27% for LT gene; half of those contains also ST gene. stx1 and stx2 genes were detected in a rate of 20.0% and 6.66%, respectively.

Few studies were held to investigate these genes in drinking water. Kaper et al. found that only 3.9% isolates were positive for at least one gene which is lower than our study; however, the eae gene was the most prevalent, as in this study [48].

In India, Ram et al. [24] found that both stx1 and stx2 genes were existing in 33.3% of isolates, and eae A gene was present in 100% of isolates.

In Brazil, out of 300* E*.* coli* isolates, five were confirmed for presence of both stx1 and stx2 genes, two for stx1, and five for stx2 only. However, none of these STEC isolates carried eae gene [49].

In another study in Bangladesh, 16 (7%) of* E*.* coli* isolates from household water supply sources owned virulence genes for EPEC and ETEC. Out of them 11 harbored LT and/or ST and only 5 had eae gene [50].

As the number of isolates was not enough to judge appropriately on the prevalence of these genes in our* E*.* coli* isolates, it is recommended to do further screening for serotypes and other virulence genes that may add more data on pathogenicity of* E*.* coli* isolated from environmental, animal, and human sources including rural areas of the city.

It could be concluded from this study that spreading the culture of antibiotic rationalization is an urgent requirement. Still, the problem of overpopulation and slums is an intractable problem which is reflected on all life aspects in poor developing countries. Another solution to decrease the rate of fecal contamination and hence diarrhea in such communities like renewal of the old water pipes in the city or building another water plant in the eastern side of city should be investigated.

## Figures and Tables

**Table 1:** PCR primer sequences, their related gene targets, and expected amplification products size.

Virulence genes	Primer sequence (5′-3′)	Product size (bp)
Heat labile toxin 1 (LT1)	F: TTACGGCGTTACTATCCTCTCTA	322
	R: CCATACTGATTGCCGCAAT	

Heat stable toxin 1 (ST1)	F: CTTTCCCCTCTTTTTAGTCAG	175
	R: TAACATGGAGCACAGGCAGG	

Shiga like toxin 1 (stx1)	F: CTGCCGGACACATAGAAGGAAACT	267
	R: AGAGGGGATTTCGTACAACACTGG	

Shiga like toxin 2 (stx2)	F: GGAGTTCAGTGGTAATACAATG	149
	R: GCGTCATCGTATACACAGG	

Intimin (eae A)	F: GAAGCCAAAGCGCACAAGACT	413
	R: CTCCGCGGTTTTAGCAGACAC	

**Table 2:** Distribution of noncoliforms, coliforms, and *E. coli* among the three districts.

District	Total number of samples	Total isolates	Noncoliforms	Total coliforms	*E. coli*
		Number (%)	Number (%)	Number (%)	Number (%)
First district	90	30 (33.3)	10 (11.1)	20 (22.2)	9 (10.0)
Second district	120	38 (31.66)	22 (18.3)	16 (13.3)	7 (5.83)
Third district	90	6 (6.66)	6 (6.66)	0 (0.0)	0 (0.0)

Total	300	74 (24.66)	38 (12.66)	36 (12.0)	16 (5.33)

**Table 3:** Distribution of positive coliform and *E. coli* isolates among seasons of the year.

Seasons	Spring	Other seasons	Test of significance	*P* value	Sig.
Number/percentage of coliform positive samples	26 (17.1%)	10 (6.75%)	*χ* ^2^ = 3.84	*P* = 0.05	S.
Number/percentage of *E. coli* positive samples	14 (9.2%)	2 (1.35%)	*χ* ^2^ = 4.59	*P* = 0.03	S.

**Table 4:** Virulence genes among *E. coli* isolates in both districts.

Gene	Total (*n* = 15)		First district (*n* = 8)		Second district (*n* = 7)	
	Positive	%	Positive	%	Positive	%
LT gene	4	26.27	2	25.0	2	28.57
ST gene	2	13.13	0	0.00	2	28.57
stx1 gene	3	20.0	2	25.0	1	14.28
stx2 gene	1	6.66	1	12.5	0	0
eae gene	5	33.33	3	37.5	2	28.57
